# Conversion to Secondary Progressive Multiple Sclerosis: Patient Awareness and Needs. Results From an Online Survey in Italy and Germany

**DOI:** 10.3389/fneur.2019.00916

**Published:** 2019-08-22

**Authors:** Alessandra Solari, Ambra Mara Giovannetti, Andrea Giordano, Carla Tortorella, Valentina Torri Clerici, Giampaolo Brichetto, Franco Granella, Alessandra Lugaresi, Francesco Patti, Marco Salvetti, Ilaria Pesci, Eugenio Pucci, Diego Centonze, Maura Chiara Danni, Simona Bonavita, Diana Ferraro, Antonio Gallo, Alberto Gajofatto, Viviana Nociti, Luigi Grimaldi, Monica Grobberio, Roberta Lanzillo, Rachele Di Giovanni, Silvia Gregori, Alessia Manni, Erika Pietrolongo, Sarah Bertagnoli, Marco Ronzoni, Laura Compagnucci, Roberta Fantozzi, Beatrice Allegri, Sebastiano Arena, Maria Chiara Buscarinu, Loredana Sabattini, Maria Esmeralda Quartuccio, Elena Tsantes, Paolo Confaloneri, Andrea Tacchino, Insa Schiffmann, Anne Christin Rahn, Ingo Kleiter, Michele Messmer Uccelli, Anna Barabasch, Christoph Heesen, the ManTra Project

**Affiliations:** ^1^Unit of Neuroepidemiology, Fondazione IRCCS Istituto Neurologico Carlo Besta, Milan, Italy; ^2^Multiple Sclerosis Unit, Fondazione IRCCS Istituto Neurologico Carlo Besta, Milan, Italy; ^3^Department of Psychology, University of Turin, Turin, Italy; ^4^Department of Neurosciences, San Camillo Forlanini Hospital, Rome, Italy; ^5^Rehabilitation Centre, Italian Multiple Sclerosis Society, Genoa, Italy; ^6^Scientific Research Area, Italian Multiple Sclerosis Foundation, Genoa, Italy; ^7^Neurosciences Unit, Department of Medicine and Surgery, University of Parma, Parma, Italy; ^8^UOSI Riabilitazione Sclerosi Multipla, IRCCS Istituto delle Scienze Neurologiche di Bologna, Bologna, Italy; ^9^Dipartimento di Scienze Biomediche e Neuromotorie, Università di Bologna, Bologna, Italy; ^10^Sezione Neuroscienze, Dipartimento di Scienze Mediche e Chirurgiche e Tecnologie Avanzate “G.F. Ingrassia”, Università di Catania, Catania, Italy; ^11^Centro Sclerosi Multipla, PO Policlinico “G. Rodolico”, Catania, Italy; ^12^IRCCS Neuromed, Pozzilli, Italy; ^13^Department of Neurosciences, Mental Health, and Sensory Organs (NESMOS), Sapienza University of Rome, Rome, Italy; ^14^Unità di Neurologia, Centro Sclerosi Multipla, Ospedale di Vaio, Fidenza, Italy; ^15^UOC Neurologia, Ospedale “A. Murri”, ASUR Marche-AV4, Fermo, Italy; ^16^Multiple Sclerosis Clinical and Research Unit, Department of Systems Medicine, Tor Vergata University, Rome, Italy; ^17^Clinica Neurologica, Università Politecnica delle Marche, Ancona, Italy; ^18^II Clinica Neurologica, Università degli Studi della Campania “Luigi Vanvitelli”, Naples, Italy; ^19^Department of Biomedical, Metabolic and Neurosciences, University of Modena and Reggio Emilia, Modena, Italy; ^20^I Clinica Neurologica, Università degli Studi della Campania “Luigi Vanvitelli”, Naples, Italy; ^21^Dipartimento di Neuroscienze, Biomedicina e Movimento, Università di Verona, Verona, Italy; ^22^Istituto di Neurologia, Fondazione Policlinico Universitario “A. Gemelli” IRCCS, Rome, Italy; ^23^UOC Neurologia e Centro Regionale SM, Fondazione Istituto “G. Giglio”, Cefalù, Italy; ^24^Laboratory of Clinical Neuropsychology, Department of Neurology and Psychology, ASST Lariana, Como, Italy; ^25^Neurosciences, Reproductive and Odontostomatological Sciences Department, Federico II University of Naples, Naples, Italy; ^26^Rehabilitation Unit, Mons. L. Novarese Hospital, Moncrivello, Italy; ^27^UOC Neurologia, Ospedale San Camillo de Lellis, Rieti, Italy; ^28^Department of Basic Medical Sciences, Neurosciences and Sense Organs, Aldo Moro University of Bari, Bari, Italy; ^29^Department of Neuroscience, Imaging and Clinical Sciences, G. D'Annunzio University of Chieti-Pescara, Chieti, Italy; ^30^Italian Multiple Sclerosis Society, Mantua, Italy; ^31^Centro Sclerosi Multipla, Ospedale “G. Salvini”–ASST Rhodense, Garbagnate Milanese, Italy; ^32^Institute of Neuroimmunology and Multiple Sclerosis, University Medical Center Hamburg-Eppendorf, Hamburg, Germany; ^33^Department of Neurology, University Medical Center Hamburg-Eppendorf, Hamburg, Germany; ^34^Kempfenhausen Centre for Treatment of Multiple Sclerosis, Marianne-Strauß-Klinik, Berg, Germany; ^35^Italian Multiple Sclerosis Society Research Foundation (FISM), Genoa, Italy

**Keywords:** multiple sclerosis, conversion, secondary progressive multiple sclerosis, online survey, patient needs, patient-physician communication

## Abstract

**Background:** Few studies have investigated the experiences of patients around the conversion to secondary progressive multiple sclerosis (SPMS). ManTra is a mixed-method, co-production research project conducted in Italy and Germany to develop an intervention for newly-diagnosed SPMS patients. In previous project actions, we identified the needs and experiences of patients converting to SPMS via literature review and qualitative research which involved key stakeholders.

**Aims:** The online patient survey aimed to assess, on a larger and independent sample of recently-diagnosed SPMS patients: (a) the characteristics associated to patient awareness of SPMS conversion; (b) the experience of conversion; (c) importance and prioritization of the needs previously identified.

**Methods:** Participants were consenting adults with SPMS since ≤5 years. The survey consisted of three sections: on general and clinical characteristics; on experience of SPMS diagnosis disclosure (aware participants only); and on importance and prioritization of 33 pre-specified needs.

**Results:** Of 215 participants, those aware of their SPMS diagnosis were 57% in Italy vs. 77% in Germany (*p* = 0.004). In both countries, over 80% of aware participants received a SPMS diagnosis from the neurologist; satisfaction with SPMS disclosure was moderate to high. Nevertheless, 28–35% obtained second opinions, and 48–56% reported they did not receive any information on SPMS. Participants actively seeking further information were 63% in Germany vs. 31% in Italy (*p* < 0.001).

Variables independently associated to patient awareness were geographic area (odds ratio, OR 0.32, 95% CI 0.13–0.78 for Central Italy; OR 0.21, 95% CI 0.08–0.58 for Southern Italy [vs. Germany]) and activity limitations (OR 7.80, 95% CI 1.47–41.37 for dependent vs. autonomous patients).

All pre-specified needs were scored a lot or extremely important, and two prioritized needs were shared by Italian and German patients: “physiotherapy” and “active patient care involvement.” The other two differed across countries: “an individualized health care plan” and “information on social rights and policies” in Italy, and “psychological support” and “cognitive rehabilitation” in Germany.

**Conclusions:** Around 40% of SPMS patients were not aware of their disease form indicating a need to improve patient-physician communication. Physiotherapy and active patient care involvement were prioritized in both countries.

## Introduction

About 15 years after clinical onset, around half of the patients with relapsing-remitting multiple sclerosis (RRMS) have developed secondary progressive disease (SPMS). This disease form is characterized by irreversible disability progression that is independent of a relapse, although patients with SPMS can still experience relapses (1, 2). Conversion from RRMS to SPMS is considered a key determinant of long-term disease prognosis (2). However, neither imaging criteria nor biomarkers are available to objectively distinguish RRMS from SPMS. SPMS is diagnosed retrospectively (3–5), and the period of diagnostic uncertainty may last for several years (3 years on average) as reported in a retrospective cohort study including 123 patients (6).

The RRMS–SPMS transition is also critical from the psychosocial point of view (7). As well as new uncertainty, people with SPMS, their families and health professionals (HPs) involved in patient care all have to adjust to the new reality of unremitting symptoms and activity limitations, and scarcity of effective disease modifying treatments (DMTs) for this disease form (8).

From a review of the literature, we found four studies on the experiences of patients around the RRMS–SPMS transition (9–12). All employed qualitative research and were conducted in the United Kingdom. Davies et al. identified four main themes envisaged by patients and carers: “realization” of the conversion to SPMS, “reaction” to this realization, “realities” of living with SPMS (dealing with the healthcare system during this period), and “future challenges” (9). The same group also explored the experiences of HPs supporting patients during the transition (10). Two main themes were found: “transition” which comprised issues related to recognizing and communicating about SPMS and “providing support” which included descriptions of challenging aspects of patient care, namely support for caregivers, multidisciplinary care and service limitations (10). Hourihan identified five themes from patient experiences: naming of the process of change, psychological consequences, consequences to occupations, impact on relationships, and coping with a life of change (11). Finally, O'Loughlin et al. focused on patient and HP experiences, and suggested a process of moving from uncertainty toward confirmation of patient's diagnostic label, the experience of which was moderated by HP attitudes and approaches (12).

Managing the Transition to SPMS (ManTra) is a mixed-method project conducted in Italy and Germany that adheres to the Medical Research Council framework for developing and evaluating complex interventions (13). The project goals were 2-fold: to assess the experiences and the needs of people who recently converted to SPMS, using qualitative and quantitative research and involving key stakeholders; and to set up a user-led resource to empower and improve the quality of life and autonomy of newly diagnosed patients with SPMS (14).

In previous ManTra project actions (paper in preparation), we identified the needs of patients converting to SPMS via literature review and a qualitative study (personal semi-structured interviews with recently diagnosed SPMS patients; focus group meetings with patient significant others, neurologists and other HPs). The present paper reports the results of an online patient survey which was conducted in Italy and Germany to: (1) assess the experiences of people recently diagnosed with SPMS; (2) verify whether the 33 needs identified in the qualitative study which involved key stakeholders are pertinent to a larger sample of people with SPMS. An additional aim (3) originated from the qualitative study, and consisted in exploring the characteristics associated to patient awareness of his/her conversion.

## Materials and Methods

### Participants

The ManTra project was approved by the ethics committees of the Fondazione IRCCS Istituto Neurologico Carlo Besta (clearance number: 27), the G D'Annunzio University of Chieti-Pescara (clearance number: 19), the Aldo Moro University of Bari (clearance number: 98793CE) in Italy, and of the Hamburg Chamber of Physicians (clearance number: PV5733) in Germany.

Patient inclusion criteria were the following: age ≥18 years; diagnosis of SPMS 3 months to 5 years before inclusion; fluent in Italian/German; informed consent provided (online or written depending on participation mode, see below). Patients unable to communicate effectively and patients with severe cognitive compromise (referring neurologist's judgment) were excluded. To increase the external validity of the study, eligible patients who could not complete the online survey were offered a telephone interview (Italy) or paper administration (Germany).

We planned to close the survey after 2 months or after 250 patients had contributed, and to extend enrolment by another 4 months to obtain a minimum number of 180 survey participants. To increase the external validity of the study, in Italy the MS centers (around 250) were invited to participate via the MS Study Group of the Italian Neurological Society. In Germany the survey was advertised during medical consultations in the MS day hospital of the Institute of Neuroimmunology and Multiple Sclerosis (INIMS) in Hamburg and the Marianne Strauss Klinik in Berg (Southern Bavaria), as well as via personal invitation letters (two rounds) to eligible patients followed at the MS day hospital of the INIMS.

### Survey Structure and Contents

The online survey consisted of an introduction and consent page, and of three questionnaires, presented in the following order: questionnaire 1—on general and clinical patient characteristics; questionnaire 2 (adapted from the Comunicazione medico-paziente nella Sclerosi Multipla, COSM) (15, 16)—on the experience of the SPMS diagnosis disclosure; questionnaire 3—on the importance and prioritization of 33 pre-specified needs identified in the ManTra qualitative study (14), and with an open section for additional needs; an open section for comments on the survey (Supplementary File 1). The surveys were identical in Italy and Germany, except for the patient determined disability scale (PDDS) (17), which was embedded in questionnaire 1 in Germany only. The survey was devised in Italian, and then translated into German.

From the ManTra qualitative study it emerged that only 40% of the patients who had converted to SPMS in the preceding 5 years were aware of their conversion, thus we changed the original (static) structure of the online survey into an adaptive one. This change and the new sample size estimation were major amendments to the study protocol (14). Specifically, two filter questions were added at the end of questionnaire 1: “Have you heard about SPMS before participating in this survey?” and “Do you have SPMS?” Patients answering “no” to the first filter question were not presented the second filter question and questionnaire 2. Patients answering “no”/”don't know” to filter question 2 were not presented questionnaire 2. Finally, the introduction of questionnaire 3 differed based on response to the filter questions (Supplementary File 1).

Before fielding the survey, its usability and functionality was tested on five Italian patients with SPMS. The online survey fulfilled the Checklist for Reporting Results of Internet E-Surveys (CHERRIES) criteria (18).

### Procedure

The MS neurologist identified potentially eligible SPMS patients and invited them to participate in the survey by contacting via email or telephone the ManTra coordinating unit (Italy) or the INIMS (Germany). Eligible patients were given the credentials to access the dedicated website (Survey Monkey in Italy; Unipark in Germany). Both platforms were password protected to ensure patient data protection and also to prevent patients from completing the questionnaires more than once. No incentives (monetary or non-monetary) were offered to survey participants. In addition, the MS neurologist provided the ManTra coordinating unit the following information for each survey participant: Expanded Disability Status Scale (EDSS) score (19), age at MS diagnosis, age at SPMS diagnosis.

### Data Analysis

The original sample size estimation based on the COSM-S section 2 score (14) was revised, based on the results of the ManTra qualitative study. We estimated (one-sample comparison) that 171 patients are sufficient to detect, with a power of 0.80, a proportion of 0.40 patients not aware of their SPMS diagnosis, compared to a hypothesized value of 0.30, at a two-sided alpha level of 0.05.

Variables were summarized using both counts and percentages, mean with standard deviation (SD), or median with minimum and maximum. Categorical variables were compared using the chi-square or the Fisher's exact test, and continuous variables using unpaired *t*-test or Wilcoxon rank-sum test (between-group comparisons), and paired *t*-test or Wilcoxon matched-pairs signed-ranks test (within-group comparisons) as appropriate. The normality assumption was tested using the Shapiro–Wilk's test.

Agreement of patient- and neurologist-reported age at MS diagnosis and age at SPMS diagnosis was assessed with the intra-class correlation coefficient (ICC) for continuous data (20), and 95% confidence intervals (CI). Absolute-agreement ICCs were calculated using mixed effects models. Bland-Altman (difference) plot were also applied to look for any systematic bias, and to identify possible outliers (21).

The contributions of potentially explanatory variables to patient's awareness of conversion to SPMS was assessed using logistic regression, calculating crude and adjusted odds ratios (ORs), and 95% CI. The independent variables were: patient age (years); gender; education (two categories: primary or secondary; university degree or higher); geographic area (four categories: Germany, North/Center/South Italy); time from SPMS conversion (years; physician-determined); EDSS (physician-determined); and activity limitations (three categories: fully autonomous, partially dependent, fully dependent). Goodness of fit of the logistic model was assessed using the Hosmer-Lemeshow test. Sensitivity analysis was performed after exclusion of SPMS patients who transitioned from more than 5 years in Italy (protocol deviations) (14).

All analyses were performed with the Stata Statistical Software, release 12.0 (Stata Corp LP, College Station, USA).

## Results

Between July and December 2018, 215 patients participated in the survey, 141 in Italy and 74 in Germany. Survey acceptability was satisfactory in Italy, with few participants abandoning the survey after reading the information (23/164, 14%); in Germany figures were higher, with 85/159 (53%) of the invited patients not participating in the survey. Missing data were few: 14 patients did not report age at SPMS diagnosis, four patients skipped the item on activity limitations, one patient the item on work status, and one patient the item on living conditions. Data quality check (logical and coherence of patient-provided information and of patient and neurologist-provided information) was satisfactory. Overall, 39% of the participants provided comments (64/141 in Italy, 19/74 in Germany) which were positive in 92%.

The 25 Italian centers were from the three geographical areas (Table 1); 11 (44%) were hospitals, 9 (36%) university hospitals, three (12%) research hospitals, and two (8%) outpatient centers. Mean patient age was 52.7 years (SD 8.7), 143 (67%) were women, and 55 (26%) had university degree or higher. Median EDSS score was 6.0 (range 2.0–9.0). Mean age at MS diagnosis was 35.2 years (SD 10.5), and mean age at SPMS diagnosis 49.8 years (SD 8.9). Table 1 show participant characteristics by country and by awareness of conversion to SPMS.

**Table 1:** Characteristics of participants to the online survey by country and by awareness of conversion to secondary progressive multiple sclerosis (SPMS).

	**Italy (*****n*** **=** **141)**			**Germany (*****n*** **=** **74)**		
**Characteristic**	**Aware (*n* = 81)**	**Not aware/unsure (*n* = 60)**	***P*-value**	**Aware (*n* = 57)**	**Not aware/unsure^c^ (*n* = 17)**	***P*-value**
	***N*** **(%)**			***N*** **(%)**		
Age, years^a^	52.3 (0.9) 37–72	50.8 (1.1) 34–72	0.29	54.5 (8.7) 36–80	54.8 (11.7) 42–84	0.57
Women	38 (63)	53 (65)	0.86	38 (67)	14 (82)	0.21
**Education (years)**						
Primary school (8)	15 (19)	17 (28)		9 (16)	0	
Secondary school (13)	40 (49)	27 (45)		30 (53)	14 (82)	
University degree or higher (16+)	26 (32)	16 (27)	0.79	18 (32)	3 (18)	0.08
**Work**						
Retired (disability)	27 (33)	17 (28)		15 (27)	3 (18)	
Full time	20 (25)	14 (23)		2 (4)	3 (18)	
Part-time	14 (17)	7 (12)		11 (20)	2 (12)	
Housewife	12 (15)	6 (10)		2 (4)	1 (6)	
Unemployed	3 (4)	9 (15)		2 (4)	0	
Retired (age)	3 (4)	2 (3)		22 (39)	7 (41)	
Other	2 (2)	5 (8)	0.04	2 (4)	1 (6)	0.43
**Status**						
Married/cohabiting	56 (69)	43 (72)		41 (73)	9 (60)	
Single	12 (15)	10 (17)		7 (12)	4 (24)	
Widow/widower	8 (10)	4 (7)		7 (12)	4 (24)	
Separated/divorced	5 (6)	3 (5)	0.89	1 (2)	0	0.36
**Country area (centers in Italy)**						
North (12)	45 (56)	25 (42)		55 (96)	15 (88)	
Center (8)	22 (27)	19 (32)		0	0	
South (5)	14 (17)	16 (27)	0.22	2 (4)	2 (12)	0.22
**Age at MS diagnosis^a^**						
Patient reported	35.4 (10.4) 17–62	33.7 (10.3) 14–61	0.92	34.0 (13.3) 14–69	33.2 (13.6) 19–72	0.60
Neurologist reported	36.5 (10.2) 20–61	34.0 (9.4) 15–64	0.14	35.3 (11.3) 19–70	31.5 (12.7) 19–72	0.29
**Age at SPMS diagnosis^a^**						
Patient reported	45.7 (8.7) 25–66	n.a.		50.7 (8.1) 37–75	n.a.	
Neurologist reported	48.8 (8.0) 33–65	47.9 (8.6) 30–72	0.51	52.4 (9.1) 32–77	53.1 (11.4) 38–79	0.81
EDSS^b^	6.0, 1.0–8.5	6.0, 3.0–7.0	0.25	6.0, 2.0–9.0	6.0, 3.0–8.5	0.31
**Patient reported activity limitations**						
Fully autonomous	18 (22)	21 (35)		14 (26)	3 (19)	
Partially dependent	54 (67)	38 (63)		35 (65)	11 (69)	
Fully dependent	9 (11)	1 (2)	0.04	5 (9)	2 (12)	0.83
**PDDS^b^**				6.0, 5.0–8.0	5.0, 5.0–8.0	0.46
**Mode of participation**						
Online (web survey)	63 (78)	40 (67)		32 (56)	12 (71)	
Telephone interview	18 (22)	20 (33)		n.a.	n.a.	
Paper	n.a.	n.a.	0.14	25 (44)	5 (29)	0.22

EDSS, expanded disability status scale; PDDS, patient determined disability scale; n.a., not applicable.

a Mean (SD) minimum—maximum.

b Median, minimum—maximum.

c *In the German sample there were the following missing data: 14 for age at SPMS diagnosis (patients who did not remember it), 4 for activity limitations, 1 for work and status*.

Overall, 138 participants (64%) were aware of their conversion to SPMS. Figures differed across the countries, with 57% of aware patients in Italy vs. 77% in Germany (*p* = 0.004). One Italian patient aware of his conversion did not remember when it occurred; corresponding figures in Germany were 14/57 (25%). Twenty one participants (12 in Italy, 9 in Germany) had SPMS since more than five years.

### Experience of Conversion to SPMS

Table 2 reports findings on the adapted COSM questionnaire in the two countries. The neurologist was the person who communicated the SPMS diagnosis to more than 80% of patients. Satisfaction with SPMS disclosure was moderate to high in both countries. Nevertheless, some 28–35% participants obtained one or more second opinions, and 48–56% reported that they did not receive any information on their disease form. Those who received information were moderately to a lot satisfied for information completeness, while satisfaction for information understandability was higher in Germany (*p* = 0.05). The proportion of those who actively sought further information about SPMS was also higher in Germany (63%) than Italy (31%; *p* < 0.001), the web being the main information source in both countries. Besides neurologists, the most reported HPs who followed the patients after SPMS diagnosis were physiotherapists in both countries, followed by rehabilitation physicians, psychologists and urologists in Italy, and by occupational therapists and psychologists in Germany.

**Table 2:** Patient self-assessed experience of the secondary progressive multiple sclerosis (SPMS) diagnosis disclosure (section 1, five items) and following period.

**Characteristic**	**Italy (*n* = 81)**	**Germany (*n* = 57)**	***P*-value**
	***N*** **(%)**		
**Conversion to SPMS disclosed by:**			
Neurologist	66 (81)	47 (82)	
Personal medical report	10 (12)	2 (4)	
Other specialist	2 (2)	6 (11)	
Other^a^	3 (4)	2 (4)	0.08
**Information on SPMS received from^b^**:			
Neurologist	37 (46)	20 (35)	
National MS Society	7 (9)	0	
Other specialist	4 (5)	9 (16)	
Another person with MS	3 (4)	0	
General practitioner	2 (2)	3 (5)	
Other^c^	4 (5)	0	
Not received	39 (48)	32 (56)	0.35
**Information understandable:**			
Not at all	0	0	
A little	5 (12)	2 (8)	
Moderately	20 (48)	6 (25)	
A lot	15 (36)	13 (54)	
Extremely	2 (5)	3 (13)	**0.05**
**Information complete:**			
Not at all	2 (5)	0	
A little	9 (21)	3 (13)	
Moderately	16 (38)	10 (42)	
A lot	12 (29)	9 (38)	
Extremely	3 (7)	2 (8)	0.24
**Searched information from other sources^b^**:			
Internet	21 (26)	32 (56)	
Magazines/journals	4 (5)	4 (7)	
Other^d^	2 (2)	1 (2)	
No	56 (69)	21 (37)	**<0.001**
**Satisfied with disclosure (replied 67/81, 83% in Italy; 49/57, 86% in germany):**			
Not at all	1 (2)	0	
A little	11 (16)	7 (14)	
Moderately	27 (40)	18 (37)	
A lot	20 (30)	19 (39)	
Extremely	8 (12)	5 (10)	0.52
**Consultation for second opinion (80/81 replies in Italy, 99%):**			
Yes	22 (28)	20 (35)	
No	58 (72)	37 (65)	0.34
Followed by other HPs (80/81 replies in Italy, 99%)^b^:	At center Out of center	At center Out of center	
Physiotherapist	8 (10) 12 (15)	1 (2) 10 (18)	
Physiatrist	8 (10) 5 (6)	1 (2) 2 (4)	
Psychologist	7 (9) 3 (4)	1 (2) 3 (5)	
Urologist	5 (6) 5 (6)	1 (2) 5 (9)	
Occupational therapist	2 (2) 0	1 (2) 7 (12)	
Other^e^	2 (2) 7 (9)	1 (2) 1 (14)	
No	57 (71)	40 (70)	0.98

Adapted from the Comunicazione medico-paziente nella Sclerosi Multipla, (COSM) questionnaire (15, 16) HP, health professional.

a Nurse (one in Italy, two in Germany), publication of the Italian MS Society, other publication.

b Totals exceed 81/57 as more than one option can be selected.

c Relative (n = 2), friend, nurse.

d TV, another person with MS (one in Italy, one in Germany).

e From MS center: Speech therapist (n = 1 Germany) neuropsychologist (n = 1 Italy), sexologist (n = 1 Italy). Out of center: Speech therapist (n = 1 Italy, n = 1 Germany), gastroenterologist (n = 1 Italy), gynecologist (n = 1 Italy), ophthalmologist (n = 1 Italy), orthopedic (n = 1 Italy), proctologist (n = 1 Italy), vascular surgeon (n = 1 Italy).

*Bold values are those statistically significant*.

### Reliability of Age at Conversion to SPMS

Agreement between patients and neurologists was excellent for age at MS diagnosis (200 observations; ICC 0.91; 95% CI 0.88−0.93; *p* < 0.001), while it was moderate for age at SPMS transition (123 observations; ICC 0.69; 95% CI 0.54−0.79; *p* < 0.001).

**Figure 1:**
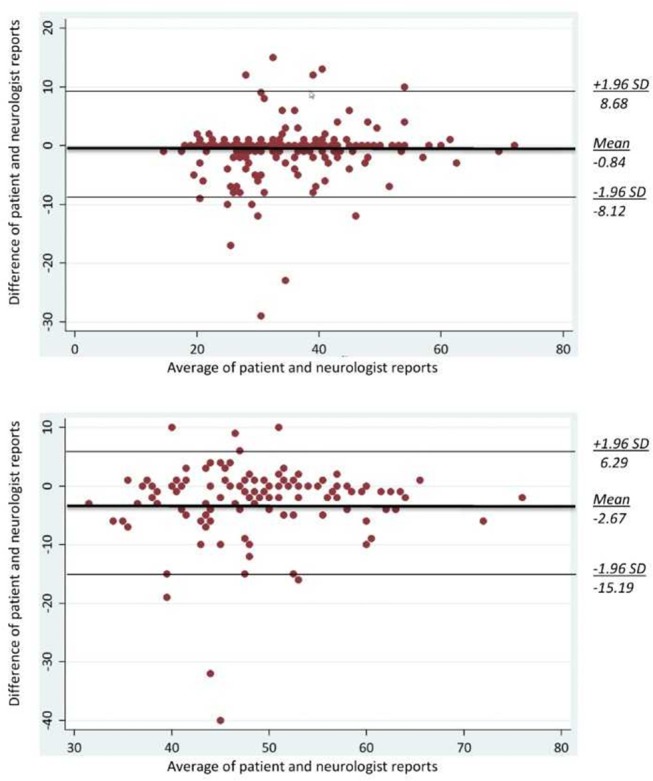
Bland-Altman plots for age at MS diagnosis (upper graph; *n* = 200) and age at SPMS diagnosis (lower graph; *n* = 81). The middle bold line is the average difference between the patient and neurologist reports. Two additional lines are the upper and lower bounds of the limits of agreement (21).

The Bland-Altman plot revealed that patients systematically reported an earlier age at SPMS conversion than neurologists (on average 2.7 years earlier; Figure 1, lower graph). Six per cent of the differences were out of the limits of agreement for both age at MS diagnosis (12/200) and age at SPMS transition (7/123). In no instance the differences seemed to be affected by the magnitude of the average age (at MS diagnosis, and at SPMS transition, respectively).

### Predictors of Patient Awareness of SPMS Conversion

In univariate analysis, the only variable associated to patient awareness of SPMS conversion was the geographic area. There was a north-south gradient (from 77 to 64%, 54%, 47%) and the OR was 0.35 (95% CI 0.15–0.78) for patients from Central Italy and 0.26 (95% CI 0.11–0.64) for patients from Southern Italy vs. Germany (reference category; Table 3). The multivariate model confirmed the independent effect of geographic area: OR 0.38 (95% CI 0.16–0.90) for Central Italy, OR 0.25 (95% CI 0.10–0.63) for Southern Italy (vs. Germany). In addition, patient self-determined activity limitations had an independent effect, with patients fully dependent having a higher odds of being aware of their SPMS conversion compared to those fully autonomous (reference category; OR 6.20, 95% CI 1.22–31.43; Table 3). The goodness of fit of the logistic model was satisfactory, and the sensitivity analysis performed after exclusion of 21 patients who transitioned from more than five years produced consistent findings (Supplementary Table 1).

**Table 3:** Variables associated with patient awareness of conversion to secondary progressive multiple sclerosis (SPMS).

**Characteristic**	**At risk *n***	**Events *n* (%)**	**Crude OR (95% CI)**	***P*-value**	**Adjusted OR (95% CI)^a^**	***P*-value**
Age, years	215	n.a.	1.02 (0.99–1.05)	0.22	1.00 (0.97–1.05)	0.77
**Sex**						
Men	72	47 (65)	1		1	
Women	143	91 (64)	0.93 (0.51–1.68)	0.81	0.88 (0.46–1.66)	0.68
**Education**						
Primary or secondary	160	99 (62)	1		1	
University degree or higher	55	39 (71)	1.50 (0.77–2.92)	0.23	1.67 (0.84–3.29)	0.15
**Geographic area**						
Germany (2 centers)	74	57 (77)	1		1	
Italy, North (12 centers)	70	45 (64)	0.54 (0.26–1.11)	0.09	0.61 (0.28–1.34)	0.22
Italy, Center (8 centers)	41	22 (54)	**0.35 (0.15**–**0.78)**	**0.01**	**0.32 (0.13**–**0.78)**	**0.01**
Italy, South (5 centers)	30	14 (47)	**0.26 (0.11**–**0.64)**	**0.03**	**0.21 (0.08**–**0.58)**	**0.003**
Time from SPMS diagnosis, years	215	n.a.	1.03 (0.94–1.12)	0.56	1.10 (0.98–1.23)	0.10
EDSS	215	n.a.	0.99 (0.80–1.23)	0.92	0.84 (0.62–1.13)	0.25
**Patient reported activity limitations**						
Fully autonomous	56	32 (57)	1		1	
Partially dependent	138	89 (65)	1.36 (0.72–2.57)	0.96	2.00 (0.88–4.53)	0.10
Fully dependent	17	14 (82)	3.50 (0.90–13.56)	0.07	**7.80 (1.47**–**41.37)**	**0.02**

CI, confidence interval; EDSS, expanded disability status scale; OR, odds ratio; n.a., not applicable.

a Multivariate model including all explanatory variables. Hosmer and Lemershow goodness-of-fit test: number of groups 10, χ_2_ 5.47, p = 0.71.

*Bold values are those statistically significant*.

### Needs Importance and Prioritization

Patients scored all the 33 pre-specified needs as a lot to extremely important, without any differences between those aware and not aware/unsure of their conversion to SPMS, and across countries (Supplementary Table 2).

The top four prioritized needs in Italy were “physiotherapy and exercise programs” (prioritized by 43% of survey participants), followed by “personalized care plan” (33%), “patient active involvement in care” (21%), and “information on social rights and policies” (17%), with a lower proportion of prioritization of the last need for patients who were aware of their SPMS conversion (Supplementary Table 2). Top prioritized needs in Germany were “physiotherapy and exercise programs” (prioritized by 40% of survey participants), followed by “patient active involvement in care” (22%), “psychological support for patients” (22%), and “cognitive rehabilitation” (21%), with similar percentages between aware and not aware patients.

## Discussion

To our knowledge, this is the first survey assessing the experience of people recently diagnosed with SPMS. The qualitative study that preceded the survey was key to revise the survey structure, which was adapted to the participant awareness. Patient participation was high in Italy.

Notably, the revised structure allowed to assess the proportion of patients who were aware of their SPMS conversion. This proportion was overall low, and in part explained by the difficulty in defining this disease form (1, 6, 22). Consistently, whilst the patient-neurologist agreement on age at MS diagnosis was excellent, this was not the case for age at SPMS conversion. Patients reported an age at SPMS conversion 2.7 years lower on average than neurologists, suggesting that they identified disease progression before SPMS was medically confirmed (Figure 1). Interestingly in the study by Katz-Sand et al. the period of diagnostic uncertainty was 2.9 years, very close to the difference between patient and physician classifications in our survey (6). Nevertheless, the difference between patient- and neurologist reported age at SPMS conversion might be due to a purposeful late communication by the neurologist (e.g., to prevent patient frightening, or DMT discontinuation). In Germany the proportion of aware patients was higher than in Italy (77 vs. 57%), however 25% of the German patients who were aware did not remember at which age they transitioned to SPMS.

Formulation of a SPMS diagnosis is not straightforward, nevertheless it is a key prognostic factor that may affect decisions and planning at the health care level (e.g., DMT change or discontinuation, provision of psychosocial support, shift to a multidisciplinary care) (23–25) and at the personal level. For these reasons, a clear and effective communication to the patient is an ethical imperative. Sharing with the patient also the period of diagnostic uncertainty, which can take some years (16), can prepare the patient to receive a confirmed SPMS diagnosis, preventing unexpected and inapt disclosure. Then inherent difficulty in diagnosing transition is combined with the difficult meaning for patients and physicians further challenging the communication. Davies et al. point out that not only patients but also HPs may need support to enter into the “possible SPMS” communication (10). Finally, delayed SPMS diagnosis disclosure may hinder treatment research on patients in the transition phase.

The logistic model found that patients fully dependent in activities of daily living were at higher odds of being aware of their conversion to SPMS, indicating that they probably realized that they had achieved irreversible disability progression. It is also worth mentioning the lower awareness of patients living in southern geographic area of Italy, pointing to a need to improve communication particularly in these contexts. While the shared decision-making competencies of MS neurologists were similar in Germany and Italy (26, 27), the decision-making preferences of MS patients seem to differ between these two countries, with German patients preferring a more active role (28, 29). This difference is paralleled in the higher proportion of patients who actively searched for further information about SPMS in Germany. It is possible that both cultural factors and differences in health system organization contributed to the differences in patient awareness of their SPMS conversion across geographic areas of the present survey.

Two of the top four prioritized needs were shared by Italian and German patients: “physiotherapy and exercise programs” and “patient active involvement in health care.” The other two differed across countries: in Italy they were “an individualized health care plan” and “information on social rights and policies,” while in Germany “psychological support for patients,” and “cognitive rehabilitation” (Supplementary Table 2). As from the MS Barometer (http://www.emsp.org/wp-content/uploads/2015/08/MS-in-EU-access.pdf), access to rehabilitation varies widely across Europe: from 100% in Belgium, Czech Republic, Denmark, Germany, Nederland, Norway and Slovakia to ≤ 15% in Cyprus, Greece, Hungary, Ireland, Romania and Spain. In Germany physiotherapy is a long-term treatment for all patients with SPMS; in Italy access to outpatient physiotherapy in the public sector is limited to 40 sessions per year, and SPMS patients have to pay for additional sessions. In most European countries MS expertise is present mainly in tertiary rehabilitative centers, theoretical approaches and quality standards vary widely, as the active involvement of the patient. To improve the management of patients transitioning to SPMS it is key to enhance the evidence base on SPMS rehabilitation, chiefly integrated, multidisciplinary approaches (30). Collaborative initiatives are needed, in the form of pragmatic trials and comparative effectiveness research. Equally important is an improvement in the communication and shared decision making competences of neurologists and other MS HPs. In the qualitative research by O'Loughlin et al., the cognitive, emotional and behavioral response during the transition from RRMS to SPMS emerged as a key theme, and information provision, a sensible communication and psychological support were considered very important in this disease phase (12).

The study has some limitations. The external validity of the findings is limited by the fact that in Germany 50% of the invited patients did not participate. It is possible that more active patients took part, which could have inflated the proportion of those aware of their transition to SPMS in this country. In addition, almost all of the German participants were from the metropolitan area of Hamburg, which is not representative of Germany as a whole. Finally, we did not validate linguistically the German version of the survey.

In conclusion, the study found that over 40% of recently diagnosed SPMS patients were not aware of their disease form, pointing to the need of an improved patient-physician communication and information exchange, also during the period of diagnostic uncertainty. Notably, patients who were aware of their diagnosis were moderately to highly satisfied with the SPMS diagnosis disclosure. Activity limitations and geographic areas were variables independently associated to patient awareness. Finally, all the 33 needs identified using a qualitative approach were judged as a lot to extremely important, and two of the four prioritized needs (physiotherapy, and active patient care involvement) were shared by Italian and German patients.

The next (on going) actions of the ManTra project (developmental phase) are the outline of four resources in each country (one for each prioritized need), guided stakeholder consensus on the most important resource, which will be also refined based on stakeholder input. This will lead in each country to a user-led resource for empowering and improving the quality of life of newly diagnosed people with SPMS, to be tested for efficacy in the next phase of the project (14).

## Data Availability

The datasets generated for this study are available on request to the corresponding author.

## Ethics Statement

The ManTra project was approved by the ethics committees of the Fondazione IRCCS Istituto Neurologico Carlo Besta (clearance number: 27), the G D'Annunzio University of Chieti-Pescara (clearance number: 19), the Aldo Moro University of Bari (clearance number: 98793CE) in Italy, and of the Hamburg Chamber of Physicians (clearance number: PV5733) in Germany.

## Author Contributions

AS and AMG conceived the online survey, with contribution of AGi and CH. AS, AMG, and MM developed the Italian version. CH, IS, and AB developed the German version. CT, VT, GB, FG, AL, FP, MS, IP, EPu, DC, MD, SBo, DF, AGa, AlG, VN, LG, MG, RL, RD, SG, AM, EPi, SBe, MR, LC, RF, BA, SA, MB, LS, MQ, ET, PC, and AT contributed to patients enrolment and provided clinical information about survey participants in Italy. IS, AR, IK, AB, and CH contributed to patient's enrolment and provided clinical information about survey participants in Germany. AS, AGi, and AB analyzed the data. AS, AMG, AGi, and CH drafted the manuscript. All authors approved the final manuscript.

### Conflict of Interest Statement

AS reports grants from Fondazione Italiana Sclerosi Multipla (FISM), during the conduct of the study; personal fees from Biogen Idec, Merck Serono, Novartis, Almirall, and Excemed. CT reports personal fees from Biogen, Merck Serono, Roche, Novartis, Genzyme, and Teva. VT reports personal fees from Biogen Idec, Merck, Novartis, Teva, Genzyme, grants and personal fees from Almirall. FG reports grants, personal fees and non-financial support from Biogen, Sanofi Genzyme, personal fees and non-financial support from Merck Serono, Roche. AL reports grants and personal fees from Biogen, Merck Serono, Novartis, Roche, Sanofi/Genzyme, and Teva. FP reports personal fees from Bayer, Biogen Celgene, Merck, Novartis, Roche, Sanofi, Teva, and Almirall. MS reports grants and personal fees from Biogen, Merck, Novartis, Roche, and Sanofi. EPu reports personal fees from Sanofi/Aventis, Biogen, Merck-Serono, Teva, Novartis, and Roche. MD reports personal fees from Novartis, Merck, Sanofi. AGa reports personal fees from Sanofi, Merck-Serono, Actelion, Roche, Teva, Mylan, Biogen, and Novartis. RL reports personal fees from Biogen, Genzyme, Merck, Roche, Teva, personal fees and non-financial support from Novartis. MR reports personal fees from Biogen, Novartis, Genzyme. PC reports grants, personal fees, non-financial support and other from Novartis, grants and non-financial support from Merk, Biogen, and Teva. IK reports personal fees from Bayer Health Care, Merck, Biogen Idec, Roche, Novartis, Sanofi, grants from Diamed, grants and personal fees from Chugai. CH reports personal fees from Biogen, Genzyme, Sanofi Aventis, Merck, Novartis, and Roche. The remaining authors declare that the research was conducted in the absence of any commercial or financial relationships that could be construed as a potential conflict of interest.

## Collaborators

### ManTra Project Investigators

*Steering Committee:* Claudia Borreani, Giovanna De Luca, Andrea Giordano, Ambra Mara Giovannetti (study Co-PI), Lara Gitto, Christoph Heesen, Alessandra Solari (study PI), Valentina Torri Clerici, Maria Trojano, Michele Messmer Uccelli.

*Literature Review Panel:* Andrea Giordano, Ambra Mara Giovannetti, Andrea Fittipaldo, Sascha Köpke.

*Qualitative Analysis Panel:* Anna Barabasch, Claudia Borreani, Ambra Mara Giovannetti, Erika Pietrolongo, Insa Shiffman.

*Expert Panel:* Anna Barabasch, Andrea Giordano, Ambra Mara Giovannetti, Lara Gitto, Erika Pietrolongo, Michele Messmer Uccelli, Insa Schiffmann, Carla Tortorella, Valentina Torri Clerici.

*Online Survey Panel:* Anna Barabasch, Andrea Giordano, Ambra Mara Giovannetti, Insa Schiffmann, Michele Messmer Uccelli.

*Centers and investigators:* Fondazione IRCCS Istituto Neurologico Carlo Besta, Milan: Unit of Neuroepidemiology, Giusi Ferrari, Andrea Fittipaldo, Arianna Fornari, Andrea Giordano, Ambra Mara Giovannetti, Alessandra Solari; Unit of Neuroimmunology and Neuromuscular Diseases: Paolo Confalonieri, Ambra Mara Giovannetti, Valentina Torri Clerici; Department of Neuroscience, Imaging and Clinical Sciences, G D'Annunzio University of Chieti-Pescara, Chieti: Giovanna De Luca, Deborah Farina, Marco Onofrj, Erika Pietrolongo; Departments of Basic Medical Sciences, Neurosciences and Sense Organs, Aldo Moro University of Bari, Bari: Pietro Jaffaldano, Alessia Manni, Maria Trojano; Unit of Psychology, Foundation IRCCS Istituto Nazionale per la Cura dei Tumori, Milan: Sara Alfieri, Claudia Borreani; Italian Multiple Sclerosis Society and Research Foundation (AISM), Department of Health Services and Research, Genova: Michele Messmer Uccelli; CEIS Economic Evaluation and HTA, Università degli Studi di Roma “Tor Vergata,” Rome, Rome: Lara Gitto. Department of Neurosciences, San Camillo Forlanini Hospital, Rome, Italy: Maria Esmeralda Quartuccio, Carla Tortorella. Institute of Neuroimmunology and Multiple Sclerosis, Department of Neurology, University Medical Center Hamburg-Eppendorf, Hamburg, Germany: Anna Barabasch, Cristoph Heesen, Anne Christin Rahn, Insa Schiffmann. Kempfenhausen Center for Treatment of Multiple Sclerosis, Marianne-Strauß-Klinik, Berg, Germany: Ingo Kleiter.
