# Supplementary material for: Conversion to Secondary Progressive Multiple Sclerosis: Patient Awareness and Needs. Results From an Online Survey in Italy and Germany
Source: Front Neurol. 2019 Aug 22;10:916. doi: 10.3389/fneur.2019.00916 (PMC6713887; doi:10.3389/fneur.2019.00916)
Supplement: Supplementary file 1 [file Data_Sheet_1.PDF]

Supplementary file 1. Contents of the ManTra online survey. Blue text describes procedures/structures. In the German version questionnaire 1 also contained the Patient Determined Disease Steps (PDDS).

ManTra project – online survey

Dear Madam/Sir,

Managing the Transition to SPMS (ManTra) aims to understand needs of newly diagnosed people with secondary progressive multiple sclerosis, in order to develop a useful resource.

The project is a collaboration between the INIMS (University of Hamburg, Germany), and the Neurological Institute C. Besta (Milan, Italy). It is funded by the Italian Multiple Sclerosis Foundation.

<u>Why we ask you to participate in the study?</u>

We have recently performed personal semi-structured interviews with people with multiple sclerosis recently and three focus group meetings with patient significant others, neurologists, and other health care professionals. By now inviting other people with multiple sclerosis to participate in the present online survey, we aim to assess the needs identified via the personal interviews on a wider, independent sample.

Your participation in this survey is <u>voluntary</u>: you may choose not to participate; if you decide to participate, you may withdraw at any time.

Your responses will be kept strictly <u>confidential.</u> To help protect your confidentiality, the survey does not contain information that will personally identify you (such as your name, email address or IP address). All the data will be stored in a password-protected electronic format. The results of this survey will be used for research purposes only, and any report of this survey will not include individual information by which you could be identified.

Clicking on the "agree" button below indicates that you: have read the above information, are at least 18 years of age, and voluntarily agree to participate in this survey.

If you do not wish to participate in this survey, please decline participation by clicking on the "disagree" button.

• **agree**

• **disagree**

QUESTIONNAIRE 1

ID: generated by the coordinator

1. **Age:** (years) drop down menu
2. **Sex:** M F
3. **Completed education:** drop down menu

No level	☐	High school degree	☐
Primary school level	☐	Undergraduate degree	☐
Middle school level	☐	Graduate degree	☐
		Doctorate/PhD degree	☐

4. **Living status:** drop down menu

Single	☐	Widow/widower	☐
Married/Cohabiting	☐	Separated/Divorced	☐

5. **Age (years) at Multiple Sclerosis diagnosis:** drop down menu

6. **Current employment:** drop down menu

Full time employed	☐	Part time employed	☐
Housewife	☐	Student	☐
Retired (age)	☐	Retired (disability/ sick leave)	☐
Unemployed	☐		

Other (please specify) Box

7. **Your autonomy in daily life activities:** drop down menu

Autonomous	☐
Partial help/assistance needed	☐
Total help/assistance needed	☐

Please read carefully the following definition:

Multiple Sclerosis is a chronic disease of the central nervous system. After a variable period from diagnosis, usually years, around half of the persons with Relapsing Multiple Sclerosis develop a Secondary Progressive form.

Secondary Progressive Multiple Sclerosis is characterized by a progressive worsening of symptoms and disability, independent from a relapse. This disease form has been defined differently over time, as “Secondary Progressive form” or simply “Progressive”.

8. Have you heard about Secondary Progressive Multiple Sclerosis before participating in this survey?

Yes □ No □

If no, skip Questionnaire 2, go to Questionnaire 3B

9. Do you have Secondary Progressive Multiple Sclerosis?

Yes □ No □ Don’t know □

Unaware (if participant selects No, Do not know): skip Questionnaire 2, go to Questionnaire 3B

QUESTIONNAIRE 2

This questionnaire assesses your experience regarding when you knew you have Secondary Progressive Multiple Sclerosis, and the following months.

---

1. **How did you know** you have Secondary Progressive Multiple Sclerosis?

I knew it from… drop down menu

- ❑ My family doctor
- ❑ The neurologist
- ❑ A physician with other specialization
- ❑ A nurse
- ❑ A relative
- ❑ Another person (please specify): BOX
- ❑ My medical records
- ❑ Other (please specify): BOX

If the answer to item 1 is:

Family doctor / Neurologist / Physician with another specialization Go to item 1.b, otherwise go to question 2

Thinking about the moment you **learned from your doctor that you have a Secondary Progressive Multiple Sclerosis,** please answer the questions below.

1.b. Are you satisfied with **the way the communication took place**?

Not at all *A little* *Somehow* *A lot* *Extremely*

If the choice is Not at all/ A little,

Why did you find the communication unsatisfactory?” BOX, not compulsory

2. **At what age** did you know you have transitioned from relapsing-remitting to Secondary Progressive Multiple Sclerosis?

drop down menu + ❑ Don't know/don't remember

3. When you knew you have Secondary Progressive Multiple Sclerosis, **did you receive information** about this type of Multiple Sclerosis?

❑ NO

❑ YES - Who informed you? Multiple-choice drop down menu

❑ Family doctor

❑ Neurologist

❑ Physician with another specialization

❑ Nurse

❑ Patient association

❑ Relative

❑ Another person with Multiple Sclerosis

❑ Other (please specify): BOX

4. Did you find the information received about Secondary Progressive Multiple Sclerosis **understandable** and **clear**?

Not at all *A little* *Somehow* *A lot* *Extremely*

5. Did you find the information received about Secondary Progressive Multiple Sclerosis **complete** and **detailed?**

Not at all *A little* *Somehow* *A lot* *Extremely*

6. In that period, did you **search information** from other sources (e.g. the web, journals, TV)?

❑ YES - Which sources: BOX, not compulsory

❑ NO

7. In the period in which you knew you have Secondary Progressive Multiple Sclerosis, did you contact neurologists external to the Multiple Sclerosis Center you were followed at for a **second opinion**?

❑ YES ❑ NO

8. In the period in which you discovered you have Secondary Progressive Multiple Sclerosis, were you followed by **other health professionals** besides those who had followed you until then?

❑ NO

❑ YES

8.1 If YES, please indicate which health professionals was form your MS Centre or external

	YES, at my MS Centre	YES, out of MS centre	NO
Physiotherapist			
Physiatrist			
Urologist			
Sexologist			
Occupational therapist			
Psychologist			
Neuropsychologist			
Other			

QUESTIONNAIRE 3A

The following is a list of **needs of those involved in the transition to Secondary Progressive Multiple Sclerosis**, identified with the contribution of the patients, their family members, and Multiple Sclerosis health care professionals.

Indicate **how important** you deem each of the needs listed below, choosing the answer that best matches your opinion.

Not at all important *A little important* *A lot important* *Extremely important*

1. **A charter** (e.g. a brochure) **of the services** available at the Multiple Sclerosis Centre
2. **A personalized care plan**, detailing the scheduled visits, exams, and/or treatments
3. **Multidisciplinary care**, a comprehensive system of care delivered by a team of professionals from a range of disciplines with different but complementary skills and knowledge.
4. **Active involvement of the patient** (and his/her significant others in the care pathway (e.g. treatment decisions).
5. **Active involvement of the family doctor** in the patient's care
6. **Connection** between the patient (and his/her family or significant others) and **the German Multiple Sclerosis Association** set up by the Multiple Sclerosis Centre
7. **Home care** provided by the Multiple Sclerosis Centre
8. **Case manager,** a professional and reference person for the patient and his/her family, who coordinates and ensures a smooth care pathway
9. **Facilitated contact** with the Multiple Sclerosis Centre in case of need
10. Access to **physiotherapy and exercise programs** to train movement, mobility and coordination
11. Access to **speech therapy**, to train swallowing and speech
12. Access to **cognitive rehabilitation**, to train cognitive functions, such as memory and attention
13. Access to **occupational therapy** to increase personal autonomy in daily and working activities
14. Access to **urological rehabilitation** to train bladder functions
15. Access to **counselling for sexual problems** to improve intimacy and sexuality
16. Access to **psychological support services** for patients
17. Access to **psychological support services** for family members or significant others
18. **Self-help groups** for patients
19. **Self-help groups** for family members or significant others

20. **Informal meetings** of patients and/or family members/significant others (e.g. 'MS cafe', 'happy hour').
21. **Job placement/replacement** support
22. Improving **patients' skills** in living with the Secondary Progressive form (e.g. management of daily life, how to search for valuable information)
23. Improving Multiple Sclerosis **health professionals' skills in 'Shared Decision Making'**: the active involvement of the patient in decisions regarding his/her care
24. Improving Multiple Sclerosis health professionals' **communication and relational skills**
25. Improving Multiple Sclerosis health professionals' **competences** on the Secondary Progressive form
26. Improving **family members and significant others' skills on Secondary Progressive form** (e.g. how to help the patient in the management of daily life, in family activities, in the education and growth of children)
27. Up-to-date and scientifically proven **information on Secondary Progressive form** for patients, family members or significant others (e.g. on diagnosis, prognosis, and treatment)
28. Up-to-date and scientifically proven **information on complementary therapies** for patients, family members or significant others (e.g. lifestyle, nutrition, aids)
29. Up-to-date **information on social rights and policies** (e.g. disability, §255 ASVG) for patients, family members and significant others
30. A certified and accessible **website** where patients, family members and significant others can share solutions and information
31. **Facilitating bureaucratic procedures** (aids, social policies)
32. **Policies for patient full inclusion, accessibility and autonomy** (e.g. transport, leisure, social life)
33. **National and international** health care providers' **guidelines** on the care of patients with Secondary Progressive Multiple Sclerosis

Which of the needs you considered as "A lot important" or "Extremely important" are **the most important** for people transitioning to Secondary Progressive Multiple Sclerosis? Select up to 3 needs.

Show the list of needs scored as important/very important, from which 1 to 5 needs can be selected

In the following box, you can write **additional needs** of people transitioning to Secondary Progressive Multiple Sclerosis

BOX, not compulsory

In the following box, you can write your comments on the survey

BOX, not compulsory

If you would like to receive the results of the survey, please write down your e-mail address (it will be used only for this purpose)

Thank you for your participation.

QUESTIONNAIRE 3B

Below is a list of **needs** identified with the contribution of the patients, their family members, and Multiple Sclerosis health care professionals.

Thinking about your current situation, indicate **how you deem important** each of the 33 needs listed below, choosing the answer that best matches your opinion.

Not at all important *A little important* *A lot important* *Extremely important*

1. **A charter** (e.g. a brochure) **of the services** available at the Multiple Sclerosis Centre
2. **A personalized care plan**, detailing the scheduled visits, exams, and/or treatments
3. **Multidisciplinary care**, a comprehensive system of care delivered by a team of professionals from a range of disciplines with different but complementary skills and knowledge.
4. **Active involvement of the patient** (and his/her family/significant others) in the care pathway (e.g. treatment decisions).
5. **Active involvement of the family doctor** in the patient's care
6. **Connection** between the patient (and his/her significant others) and **the German Multiple Sclerosis Association** set up by the Multiple Sclerosis Centre
7. **Home care** provided by the Multiple Sclerosis Centre
8. **Case manager,** a professional and reference person for the patient and his/her family, who coordinates and ensures a smooth care pathway
9. **Facilitated contact** with the Multiple Sclerosis Centre in case of need
10. Access to **physiotherapy and exercise programs** to train movement, mobility and coordination
11. Access to **speech therapy**, to train swallowing and speech
12. Access to **cognitive rehabilitation**, to train cognitive functions, such as memory and attention
13. Access to **occupational therapy** to increase personal autonomy in daily and working activities
14. Access to **urological rehabilitation** to train bladder functions
15. Access to **counselling for sexual problems** to improve intimacy and sexuality
16. Access to **psychological support services** for patients
17. Access to **psychological support services** for family members or significant others
18. **Self-help groups** for patients

19. **Self-help groups** for family members or significant others
20. **Informal meetings** of patients and/or family members or significant others (e.g. 'MS cafe', 'happy hour').
21. **Job placement/replacement** support
22. Improving **patients' skills** in living with the Secondary Progressive form (e.g. management of daily life, how to search for useful information)
23. Improving Multiple Sclerosis **health professionals' skills in 'Shared Decision Making'**: the active involvement of the patient in decisions regarding his/her care
24. Improving Multiple Sclerosis health professionals' **communication and relational skills**
25. Improving Multiple Sclerosis health professionals' **competences** on the Secondary Progressive form
26. Improving **family members and significant others' skills on Secondary Progressive form** (e.g. how to help the patient in the management of daily life, in family activities, in the education and growth of children)
27. Up-to-date and scientifically proven **information on Secondary Progressive form** for patients, family members or significant others (e.g. on diagnosis, prognosis, and treatment)
28. Up-to-date and scientifically proven **information on complementary therapies** for patients, family members or significant others (e.g. lifestyle, nutrition, aids)
29. Up-to-date **information on social rights and policies** (e.g. disability, §255 ASVG) for patients, family members and significant others
30. A certified and accessible **website** where patients, family members and significant others can share solutions and information
31. **Facilitating bureaucratic procedures** (aids, social policies)
32. **Policies for patient full inclusion, accessibility and autonomy** (e.g. transport, leisure, social life)
33. **National and international** health care providers' **guidelines** on the care of patients with Secondary Progressive Multiple Sclerosis

Which of the following needs are **the most important** in your current situation?

Show the list of needs scored as important/very important, from which 1 to 5 needs can be selected

In the following box, you can write **additional needs** you consider important

BOX, not compulsory

In the following box, you can write your comments on the survey

BOX, not compulsory

If you would like to receive the results of the survey, please write down your e-mail address (it will be used only for this purpose)

Thank you for your participation.
