# Supplementary material for: Conversion to Secondary Progressive Multiple Sclerosis: Patient Awareness and Needs. Results From an Online Survey in Italy and Germany
Source: Front Neurol. 2019 Aug 22;10:916. doi: 10.3389/fneur.2019.00916 (PMC6713887; doi:10.3389/fneur.2019.00916)
Supplement: Supplementary file 2 [file Data_Sheet_2.PDF]

Supplementary table 1. Sensitivity analysis performed after exclusion of the 21 patients who converted to secondary progressive multiple sclerosis (SPMS) since > 5 years. CI, confidence interval; EDSS, expanded disability status scale; OR, odds ratio; n.a., not applicable.

**Characteristic**	**At risk n**	**Events n (%)**	**Crude OR (95% CI)**	**P value**	**Adjusted OR (95% CI)[1]**	**P value**
Age, years	194	n.a.	1.02 (0.98 - 1.06)	0.25	1.00 (0.97 - 1.05)	0.75
Sex						
Men	63	41 (65)	1		1	
Women	131	82 (63)	0.90 (0.48 - 1.68)	0.74	0.85 (0.43 - 1.66)	0.63
Education						
Primary or secondary	88	47 (53)	1		1	
University Degree or higher	39	25 (64)	1.67 (0.84 - 3.32)	0.15	1.79 (0.90 - 3.60)	0.10
Geographic Area						
Germany (2 centres)	67	51 (76)	1		1	
North (12 centres)	66	43 (65)	0.59 (0.27 - 1.25)	0.17	0.67 (0.28 - 1.57)	0.36
Centre (8 centres)	37	18 (49)	**0.30 (0.13 - 0.70)**	**0.006**	**0.28 (0.10 - 0.74)**	**0.01**
South (5 centres)	24	11 (46)	**0.26 (0.10 - 1.71)**	**0.008**	**0.23 (0.08 - 0.70)**	**0.01**
Time from SPMS diagnosis, years	127	n.a.	**2.88 (2.08 - 3.98)**	**<0.001**	1.11 (0.89 - 1.40)	0.35
EDSS	127	n.a.	0.98 (0.79 - 1.22)	0.88	0.82 (0.59 - 1.14)	0.24
Patient reported activity limitations						
Fully autonomous	54	30 (56)	1		1	
Partially dependent	120	77 (64)	1.43 (0.74 - 2.76)	0.28	2.30 (0.97 - 5.48)	0.06
Fully dependent	16	13 (81)	3.47 (0.88 - 13.63)	0.07	**8.66 (1.50 - 50.16)**	**0.02**

1. Multivariate model including all explanatory variables. Hosmer and Lemershow goodness-of-fit test: number of groups 10, χ_2 10.49, p=0.23.
