# Supplementary material for: Conversion to Secondary Progressive Multiple Sclerosis: Patient Awareness and Needs. Results From an Online Survey in Italy and Germany
Source: Front Neurol. 2019 Aug 22;10:916. doi: 10.3389/fneur.2019.00916 (PMC6713887; doi:10.3389/fneur.2019.00916)
Supplement: Supplementary file 3 [file Data_Sheet_3.PDF]

Supplementary table 2. Median (interquartile range) importance of the 33 pre-defined needs (upper table) and the top four prioritized needs (lower table) by patient awareness of secondary progressive multiple sclerosis (SPMS) conversion. Importance is rated on a 1–4 scale (not at all/a little/a lot/extremely). Of the 138 patients who completed questionnaire 3 in Italy, 124 prioritized three needs, 12 two needs, and two one need. Of the 67 patients who completed questionnaire 3 in Germany, 61 prioritized three needs, and 6 two needs. SO, significant other; HP, health professional.

Need	Italy		Germany	
	**Aware (n=79)**	**Not aware (n=59)**	**Aware (n=52)**	**Not aware (n=15)**
1. Charter of MS centre services	3 (2 – 3)	3 (2 – 3)	3 (3 – 4)	3 (2 – 3)
2. Personalized health care plan	3 (3 – 4)	3 (3 – 4)	3 (2 – 4)	3 (3 – 3)
3. Multidisciplinary care	3 (3 – 4)	3 (3 – 4)	3 (3 – 4)	3 (3 – 4)
4. Patient active involvement in health care	3 (3 – 4)	3 (3 – 4)	3 (3 – 4)	3 (3 – 4)
5. Family doctor active involvement in health care	3 (2 – 3)	3 (2 – 3)	3 (2 – 4)	3 (3 – 4)
6. Connection of patient/SO and MS Society at MS centre	3 (2 – 3)	3 (2 – 3)	3 (2 – 4)	2 (2 – 3)
7. Home care provided by the MS centre	3 (2 – 3)	3 (2 – 4)	3 (2 – 4)	3 (2 – 3)
8. Case manager	3 (3 – 3)	3 (2 – 3)	3 (3 – 4)	3 (2 – 4)
9. Facilitated contact with the MS centre	4 (3 – 4)	3 (3 – 4)	4 (3 – 4)	3 (3 – 4)
10. Physiotherapy and exercise programs	4 (3 – 4)	4 (3 – 4)	4 (4 – 4)	4 (3 – 4)
11. Speech therapy	3 (2 – 4)	3 (2 – 4)	3 (2 – 4)	3 (2 – 4)
12. Cognitive rehabilitation	3 (3 – 4)	3 (2 – 4)	4 (3 – 4)	4 (3 – 4)
13. Occupational therapy	3 (3 – 4)	3 (3 – 4)	4 (3 – 4)	4 (2 – 4)
14. Urological rehabilitation	3 (3 – 4)	3 (3 – 4)	3 (3 – 4)	4 (3 – 4)
15. Sexologist	3 (2 – 4)	3 (2 – 3)	3 (3 – 4)	3 (2 – 4)
16. Psychological support for patients	3 (3 – 4)	3 (3 – 4)	3 (3 – 4)	3 (3 – 4)
17. Psychological support for SOs	3 (2 – 3)	3 (2 – 4)	3 (3 – 4)	4 (3 – 4)
18. Self-help groups for patients	3 (2 – 3)	3 (2 – 3)	3 (2 – 4)	3 (2 – 4)
19. Self-help groups for SOs	2 (2 – 3)	3 (2 – 3)	2 (2 – 4)	2 (1 – 3)
20. Informal meetings of patients/SOs	3 (2 – 3)	2 (2 – 3)	3 (2 – 4)	2 (2 – 3)
21. Job placement/replacement support	2 (2 – 4)	2 (2 – 4)	2 (2 – 4)	2 (2 – 4)
22. Improving patient skills in living with SPMS	3 (3 – 4)	3 (3 – 4)	3 (2 – 4)	3 (1 – 3)
23. Improving MS HP shared decision making skills	4 (3 – 4)	3 (3 – 4)	4 (3 – 4)	3 (3 – 4)

24. Improving MS HP communication/relational skills	4 (3 – 4)	3 (3 – 4)	4 (3 – 4)	3 (3 – 4)
25. Improving MS HP competences on SPMS	3 (3 – 4)	3 (3 – 4)	4 (3 – 4)	4 (3 – 4)
26. Improving SO skills on SPMS	3 (3 – 4)	3 (3 – 4)	3 (2 – 4)	3 (2 – 4)
27. Information on SPMS for patients/SOs	3 (3 – 4)	3 (3 – 4)	4 (3 – 4)	4 (3 – 4)
28. Information on complementary therapies	3 (3 – 4)	3 (3 – 4)	4 (3 – 4)	4 (3 – 4)
29. Information on social rights/policies	4 (3 – 4)	3 (3 – 4)	4 (3 – 4)	3 (3 – 4)
30. Certified and accessible website	3 (3 – 4)	3 (2 – 4)	3 (3 – 4)	3 (2 – 4)
31. Facilitate bureaucratic procedures	4 (3 – 4)	4 (3 – 4)	4 (3 – 4)	4 (3 – 4)
32. Policies for patient social inclusion/accessibility	4 (3 – 4)	3 (3 – 4)	4 (3 – 4)	4 (4 – 4)
33. Guidelines for HPs caring patients with SPMS	4 (3 – 4)	3 (3 – 4)	3 (3 – 4)	4 (3 – 4)

**Prioritized needs**	**Italy**			**Germany**		
	**Aware (n=79)**	**Not aware (n=59)**	**P value**	**Aware (n=52)**	**Not aware (n=15)**	**P value**
	No (%)				***No (%)***	
10. Physiotherapy and exercise programs	37 (47)	23 (39)	0.36	21 (40)	6 (40)	1.00
4. Patient active involvement in health care	18 (23)	11 (19)	0.55	11 (21)	4 (27)	0.72
2. Personalized health care plan	27 (34)	19 (32)	0.80			
29. Information on social rights/policies	9 (11)	15 (25)	0.03			
16. Psychological support for patients				12 (23)	3 (20)	1.00
12. Cognitive rehabilitation				10 (19)	4 (27)	0.53
